# Yak-Derived CXCL14 Activates the Pro-Inflammatory Response of Macrophages and Inhibits the Proliferation and Migration of HepG2

**DOI:** 10.3390/ani13193036

**Published:** 2023-09-27

**Authors:** Biao Li, Juan Li, Li Wang, Yong Wei, Xiaolin Luo, Jiuqiang Guan, Xiangfei Zhang

**Affiliations:** 1 Key Laboratory of Qinghai-Tibetan Plateau Animal Genetic Resource Reservation and Utilization, Ministry of Education, Southwest Minzu University, Chengdu 610041, China; 18874028579@163.com (B.L.); lengya01@126.com (J.L.); 2 Key Laboratory of Animal Science of State Ethnic Affairs Commission, Southwest Minzu University, Chengdu 610041, China; 3 Sichuan Animal Sciences Academy, Chengdu 610041, China; gjq1900@163.com (J.G.); zxfsicau@gmail.com (X.Z.); 4 Sichuan Academy of Grassland Sciences, Chengdu 611731, China; luoxl2004@sina.com

**Keywords:** yak, CXCL14, macrophage, HepG2, immunity

## Abstract

**Simple Summary:**

In this study, the yak CXCL14 protein was successfully obtained through prokaryotic expression. In vitro experiments proved that the CXCL14 protein can not only significantly increase the activity of spleen macrophages and promote the expression of pro-inflammatory cell-related factors but also inhibit the activity, clone formation and migration of HepG2. This study aims to further understand the function of the CXCL14 protein in yaks.

**Abstract:**

*CXCL14* (C-X-C motif chemokine ligand 14) is an important chemokine involved in infection and immunity and plays an important role in a variety of immune-related diseases. The 446 bp cDNA sequence of the *CXCL14* gene in yaks was obtained. Additionally, the prokaryotic expression vector of the CXCL14 protein with a molecular weight of 27 kDa was successfully constructed and expressed. The proliferation activities and migration abilities of spleen macrophages were significantly inhibited after treatment with the CXCL14 protein at different concentrations (1, 10 and 20 μg/mL) (*p* < 0.05). Furthermore, the expressions of pro-inflammatory cytokines interleukin 1 beta (*IL-1β*), interleukin 6 (*IL6*), interleukin 8 (*IL8*) and interferon-α (*TNF-α*) were significantly increased (*p* < 0.05), but the expression of anti-inflammatory factor interleukin 10 (*IL10*) was significantly decreased (*p* < 0.05). The contents of inflammatory factors in the supernatant of cells were detected using ELISA, and it was also found that the contents of TNF-α, IL6 and cytochrome c oxidase subunit II (COX2) were significantly increased under different CXCL14 protein concentrations (*p* < 0.05). Finally, the exogenous addition of CXCL14 inhibited the activity, clonal formation and migration of hepatoma cells (HepG2). Additionally, after HepG2 cells were treated with 20 μg/mL CXCL14 protein for 12 h, 24 h and 36 h, the expression levels of BCL2 homologous antagonist/killer (*BAK*) and the BCL2-associated X apoptosis regulator (*BAX*) were increased to varying degrees, while the expression levels of hypoxia-inducible factor 1 subunit alpha (*HIF1A)*, the mechanistic target of rapamycin kinase (*mTOR*) and cyclin-dependent kinase 1 (*CDK1*) genes decreased compared to the control group. In conclusion, the CXCL14 protein can inhibit the proliferation and migration of HepG2 cells by inducing the expression of macrophage pro-inflammatory factors and activating apoptosis-related genes to exert innate immunity. These results are helpful to further study the function of the CXCL14 protein and provide research data for the innate immune mechanism of yaks under harsh plateau environments.

## 1. Introduction

Macrophages, as remarkable plastic immune cells, play an important role in innate and cellular immunity in mammals. Macrophage polarization is a process that exhibits a specific phenotype and responds functionally to the microenvironmental stimuli and signals encountered in each specific tissue. For example, M1 macrophages have strong anti-microbial and anti-tumor activities, while M2 macrophages can inhibit chronic inflammatory reactions through anti-inflammatory effects [1]. *CXCL14* is a highly conserved and bioactive cytokine, which is responsible for activating the infiltration and cell mobilization of immune cells, thus participating in immune responses and playing an important role in a variety of immune-related diseases [2,3]. It is not only expressed in many normal cells but has also been reported to be closely related to the occurrence of cancer-related diseases [4]. In addition, it has been reported that CXCL14 in some mammals not only has certain bactericidal activity but can also directly enhance the antibacterial function of macrophages to prevent infection caused by a variety of microorganisms [5,6]. The reason for the above function could be related to the striking common structural characteristics of a large number of antimicrobial peptides (AMPs), such as defensins and cathelicidins, including large plaques with positive charges in their molecular surfaces, three antiparallel β chains shown in β-defensins, and the typical C-terminal α helix of cathelicidin LL-37 [5,6,7]. Further, studies have found that *CXCL14*-deficient mice, when exposed to the cold, showed a decreased recruitment of macrophages and the impaired activity of brown adipose tissue, thus affecting their ability to resist cold environments [8].

Yaks (*Bos grunniens*), as one of the endemic species of the Qinghai–Tibet Plateau (QTP) at a high altitude, have strong adaptability and immunity due to the harsh plateau environment such as low temperatures, low oxygen and high ultraviolet radiation. Therefore, yaks have been a representative model animal in the study of altitude adaptation and have long been the best research object to explore the mechanism of animal adaptation to the harsh environment of low temperature and hypoxia [9]. However, there are few reports on how *CXCL14* plays the related functions of innate immunity in yaks. In this study, the CXCL14 protein of the Maiwa yak was analyzed, characterized and overexpressed in vitro, and immune-related factors of the obtained protein were studied in spleen macrophages of yak. These results provide certain reference materials for the anti-inflammatory mechanism of yaks.

## 2. Materials and Method

### 2.1. Materials

*Escherichia coli* was purchased from the China Center of Industrial Culture Collection and maintained in our laboratory (The Key Laboratory of Qinghai–Tibet Plateau Animal Genetic Resource and Utilization, Ministry of Education). This strain was cultured at 37 °C for 24 h in a nutrient broth (NB, Thermo Fisher Scientific, Shanghai, China) medium. HepG2 cells were purchased from the Cell Resource Center in IBMS and CAMS/PUMC. HepG2 cells were cultured in Eagle’s medium (DMEM, Invitrogen, Carlsbad, CA, USA) supplemented with 10% FBS (Hyclone) and 1% penicillin/streptomycin (Invitrogen, Carlsbad, CA, USA).

### 2.2. Cell Isolation and Culture

Yak samples were collected from a local farm in Hongyuan County, Sichuan Province, China. A total of 9 female yaks were classified as juvenile yaks (1 day old), young yaks (15 months old) and adult yaks (5 years old). Three biological replicates were collected from the yaks at each stage. The execution method of yaks and the preservation method of the samples are described in the previous period of our laboratory [10]. All animal experimental treatment methods were carried out in accordance with the Regulations on the Administration of Experimental Animals of the Ministry of Science and Technology of China (revised in June 2004) and were approved by the Institutional Animal Care and Utilization Committee of Southwest Minzu University, Chengdu, China. Fresh yak spleen tissue was washed with a 1% PBS buffer and cut into the DMEM medium with 10% FBS until the tissue blocks were cut into cell homogenates and filtered into a suspension using a disposable 200-mesh screen. The cells were re-suspended with 5 mL pre-cooled 1 × RBC lysate (Thermo Fisher Scientific, Shanghai, China) and impended on ice for 5 min. The cell suspension was then washed with 10 mL of pre-cooled PBS. The cells were centrifuged at 600× *g* at 4 °C for 5 min, and the supernatant was discarded. The obtained cell precipitates were re-inoculated with the RPMI 1640 medium (Sigma-Aldrich, Louis, MO, USA), and the opposing cells were washed off after 3 h of static culture at 37 °C, and the remaining adherent cells were macrophages. The cells were counted using a cell counter and inoculated into cell culture plates at a density of 2–3 × 10^6^ cells/mL for subsequent experiments.

### 2.3. RNA Isolation and qRT-PCR Analysis

The total RNA was extracted using the TRI-zol reagent (Invitrogen, Carlsbad, CA, USA) per the manufacturer’s recommendations. Nanodrop 2000 was employed to quantify the concentration of RNA (Thermo Fisher Scientific, Waltham, MA, USA). RNA preparations with an A260/A280 ratio of 1.8–2.0 and an A260/A230 ratio greater than 2.0 were used for subsequent analysis. The *CXCL14* gene was amplified using a PCR and the spleen cDNA of the adult yak as a template, and the primers were F:5′- CCCTCCCCGAAAACC-3′, R:5′-TGTCTTATGCCTGTGAGAAAG-3′. The primers were synthesized by Shengong Biotech Co., LTD. (Shanghai, China) qRT-PCR was performed with the SYBR Green master mix (Takara, Beijing, China) applying a StepOne Plus RT-PCR system (Thermo Fisher Scientific, Waltham, MA, USA). For the determination of the mRNA expression levels, the 2^–ΔΔCT^ method was applied [11]. *β-actin* was used as the reference gene, and the primer sequence was F:5′-CTTCGAGCAGGAGATGGC-3′, R:5′-CCGTGTTGGCGTAGAGGT-3′. The *CXCL14* primer sequence was F:5′-GCACTGCGAGGAGAAGATG-3′, R:5′-TCGTTCCAGGCGTTGTAC-3′. In addition, other target amplification primers are given in Table 1.

### 2.4. Bioinformatics Analysis

The homology analysis of *CXCL4* gene sequences in yaks was conducted using DNASTAR 17.1.1 software and Blast (https:/blast.ncbi.nlm.nih.gov/Blast.cgi) (accessed on 10 January 2022). MEGA7.0 was used to construct phylogenetic trees. On the other hand, ExPASy (https:/web.expasy.org/protparam) (accessed on 10 January 2022) and ProtScal (https:/web.expasy.org/protscale/) (accessed on 10 January 2022) online servers were used to measure the physicochemical properties and hydrophobicity of proteins. The functional domain analysis of the protein structure and interaction protein analysis were performed using SMART (http:/smart.embl-heidelberg.de/) (accessed on 10 January 2022). SOPMA (https:/prabi.ibcp.fr/htm/site/web/home) (accessed on 10 January 2022) and SWISS-MODEL (https:/swissmodel.expasy.org/) (accessed on 10 January 2022) online servers were used to predict the secondary and tertiary structures of the proteins, respectively.

### 2.5. Plasmid Construction, Expression, and Purification of CXCL14

The cloned yak gene *CXCL14* was digested by *Ecor I* and *Hind III* (Takara, Beijing, China), and the target fragment was cloned into pET-32a(+) vector plasmid. The expression vector was successfully constructed, and the strains were activated. The plasmid pET-32a (+) -CXCL14 was transformed into *E. coli* BL21 (DE3) and induced with isopropyl β-D-1-thiogalactoside (IPTG) at different concentrations (0.5, 1, 1.5 mmol/L). After incubation at 37 °C for 12 h, 1 mL of the bacterial solution was absorbed and centrifuged at 4000 r/min for 5 min. The supernatant was discarded and then re-suspended with a 100 μL PBS buffer solution. The protein expression was detected by 12% SDS-PAGE. A large amount of the recombinant protein was induced, and the activated bacterial solution was centrifuged at 4 °C for 20 min at 8000 r/min, and the bacterial precipitation was collected. After the bacteria were re-suspended with a PBS phosphate buffer, the bacteria were repeatedly frozen and thawed at 37 °C 3 to 5 times, centrifuged at 8000 r/min at 4 °C for 20 min; the superserum and precipitation were separated, and precipitation was collected. The precipitated target protein was purified according to the instructions of the labeled Protein Purification Kit (CWbiotech, China). The purified CXCL14 protein was detected by 12% SDS-PAGE. After quantification with the BCA Protein Assay Kit (Beyotime, Shanghai, China), the rest was stored at −80 °C.

### 2.6. Detection of Recombinant Protein Virulence

The expression bacteria of *Escherichia coli* BL21 empty plasmid and the CXCL4 recombinant protein were activated, and bacteria were adjusted to a uniform concentration when the OD_600mm_ was 0.4~0.6. After 10-fold dilution, the recombinant expression bacteria were gradually diluted for 5 gradients. The diluted bacterial solution was absorbed at 5 μL points on an LB solid medium containing ampicillin (final concentration 100 μg/mL) and IPTG (final concentration 1.0 mmol/L). The empty Escherichia coli plasmid was used as the control group and cultured overnight at 37 °C. The colony morphology and quantity of the experimental group and control group were observed, and the virulence of the CXCL14 protein against *Escherichia coli* BL21 was analyzed.

### 2.7. Cell Activity

Macrophages were inoculated into 96-well plates with 1 × 10^4^ cells per well and were treated with the CXCL14 protein at different final concentrations (1 μg/mL, 10 μg/mL and 100 μg/mL). The control group was treated with the same buffer as the protein group to eliminate the effects of the buffer. After 24 h of treatment, the cells were treated according to the instructions of the CCK8 kit (Tongren, Tokyo, Japan). The absorbance values at 450 nm were measured every half hour, and data were analyzed using SPSS 26.0 software. HepG2 cell viability was detected using the same method as above.

### 2.8. Cell Clonality and Migration

HepG2 cells were inoculated with 1 × 10^3^ cells/well in a 6-well plate and treated with the CXCL14 protein at a final concentration of 20 μg/mL. After 2 weeks of culture, the cells were fixed with 4% paraformaldehyde for 30 min, washed with sterile PBS 2 to 3 times, and stained with crystal violet for 10 min. After washing and drying, the cells were photographed and counted. SPSS 26.0 software was used to analyze data significance. HepG2 cells were inoculated with 5 × 10^5^ cells/well in a 24-well plate and cultured with DMEM medium containing 10% FBS for 24 h until the monolayer cells were grown. Single-layer hepatocellular carcinoma cells were marked with rulers and tips, washed with a PBS buffer 2 to 3 times, and added into the DMEM medium. The experimental group was treated with the CXCL14 protein at 20 μg/mL, while the control group was added using a buffer. Cells incubated for 36 h under the same conditions were taken out, and the migration of HepG2 cells was observed under an inverted microscope. The migration degree was analyzed using Image J2 software, and the data significance was analyzed using SPSS 26.0 software.

### 2.9. Statistical Analysis

All experiments were tested by one-way analysis of variance (ANOVA) using SPSS software (version 26.0 for Windows). Significant differences at *p* < 0.05 were denoted with *. Significant differences at *p* < 0.01 were denoted with **.

## 3. Result

### 3.1. Cloning and Bioinformatics Analysis of Yak CXCL14

**Figure 1:**
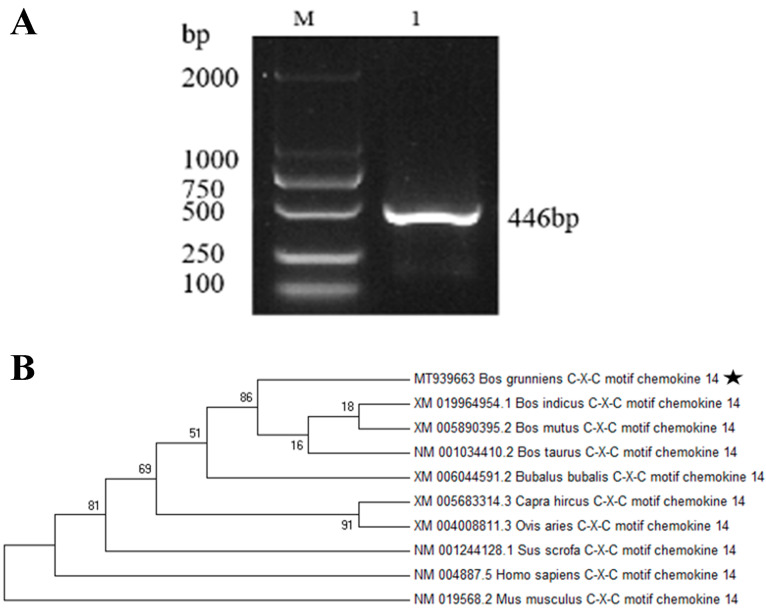
Sequence analysis of the *CXCL14* gene. (**A**) Result for PCR amplification of the *CXCL14* gene; (**B**) Phylogenetic tree of the *CXCL14* gene.

According to the cDNA of Maiwa yak spleens as a template, the 446 bp *CXCL14* chemokine gene was amplified (Figure 1A). The obtained nucleotide sequence and amino acid sequence were submitted to the GenBank database of the NCBI website (accession number: MT939663). Homology analysis showed that the *CXCL14* gene sequence of Maiwa yaks was highly conserved among the Bovis species and was clustered among wild yaks, Bos indicus and Bos taurus, with homology at 99.9%, but demonstrated the most distant relationship with mice and humans with a homology of 73.3% (Figure 1B). In addition, through the analysis of the physical and chemical properties of the amino acid sequence of the CXCL14 protein, its molecular formula was found to be C_522_H_858_N_152_O_137_S_9_, its molecular weight was 11.74 kDa, and its theoretical isoelectric point was 9.91. Among these, the negatively charged residue radix (Asp + Glu) was 9, and the positively charged residue radix (Arg + Lys) was 23, which is generally positively charged. The fat coefficient was 80.81, the half-life was 30 h, and the instability coefficient was 40.31. It can be inferred that yak CXCL14 is an unstable alkaline hydrophilic protein. The CXCL14 protein also has three secondary structures: α helix (22.29%), extended fragment (10.10%) and random coil (60.61%).

### 3.2. Prokaryotic Expression and Identification of Yak CXCL14 Protein

**Figure 2:**
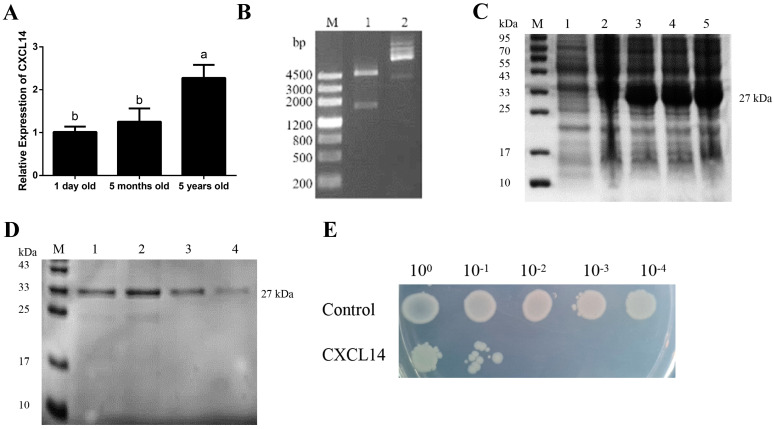
Prokaryotic expression and cytotoxicity analysis of recombinant yak CXCL14 protein. (**A**) Differential expression of *CXCL14* in spleens at different growth stages; a,b: Different letters indicate a significant level of difference; (**B**) Identification of pET-32a-CXCL14 through double enzyme digestion; (**C**) The expression results of the CXCL14 protein; M: Protein maker; 1: *E. coli* strain BL21 (DE3) with empty vector pET32a (+); 2~5: the results of CXCL14 protein induction with 0 mmol/L, 0.5 mmol/L, 1 mmol/L, 1.5 mmol/L IPTG; (**D**) The purification results of the CXCL14 protein; (**E**) The cytotoxicity of the CXCL14 protein to *E. coli*.

First, the expression of the *CXCL14* gene in Maiwa yak spleens at birth (1 day), adolescence (15 months) and adulthood (5 years old) was analyzed, and it was found that the *CXCL14* gene in the spleen of adult yaks was significantly higher than that in newborn and adolescence yaks (*p* < 0.05) (Figure 2A). In order to further explore the function of *CXCL14*, the prokaryotic expression vector of the yak CXCL14 protein was designed, and the 27 KDa CXCL14 protein was obtained after being induced by IPTG at different concentrations and detected using SDS-PAGE (Figure 2B,C). The results showed that the purified protein could be recognized and bound by a histidine-labeled antibody (Figure 2D). Moreover, we also found that the CXCL14 protein had a certain toxic effect on the host bacterium BL21(DE3). Compared with the control group, it was found that the growth of bacteria containing the recombinant vector of pET-32a-CXCL14 was inhibited, and the colonies were smaller and fewer with the increase in dilution, which indicates that the CXCL14 protein had an obvious inhibitory effect on the host Escherichia coli (Figure 2E).

### 3.3. Effects of CXCL14 Protein on Macrophages under Different Concentrations

**Figure 3:**
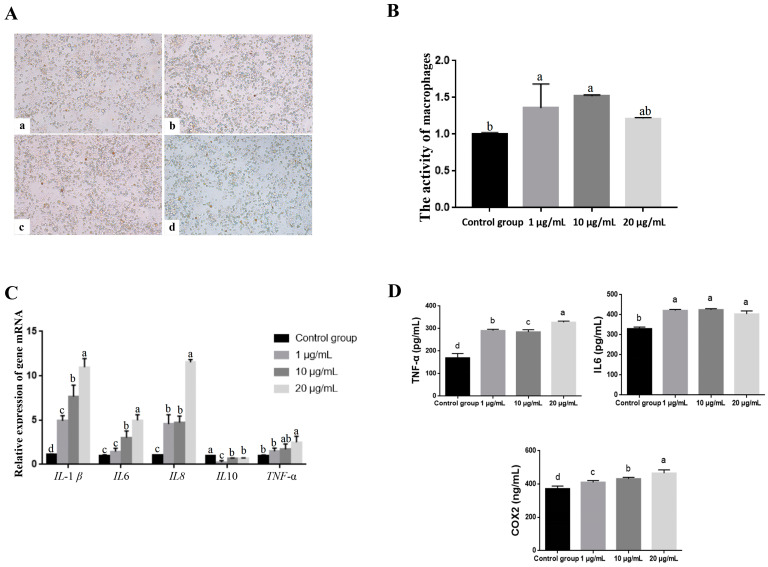
Effect of CXCL14 protein treatment on macrophages. Morphology of yak macrophages treated with the CXCL14 protein (100×); (**A**): (**a**) Control group; (**b**): 1 μg/mL; (**c**) 10 μg/mL; (**d**) 20 μg/mL; (**B**–**D**) Detection of macrophage activity and inflammatory factors using the CXCL14 protein; a,b,c,d: Different letters indicate a significant level of difference.

In order to verify the role of the CXCL14 protein in yak spleen macrophages, we further detected the activity of macrophages and the expression of related pro-inflammatory cytokines. First, different concentrations of the exogenous CXCL14 protein were added to yak macrophages, and it was found that different concentrations of the CXCL14 protein had no significant effect on cell morphology (Figure 3A). Macrophage activity was increased under the treatment of 1 μg/mL, 10 μg/mL and 20 μg/mL of the CXCL14 protein, and cell activity under the treatment of 1 μg/mL and 10 μg/mL CXCL14 protein was significantly higher than that of the control group (*p* < 0.05) (Figure 3B). In addition, RT-qPCR showed that the expressions of *IL-1β*, *IL-6*, *IL-8* and *TNF-α* in yak macrophages increased with the increase in the CXCL14 protein’s concentration and were significantly higher than those in the control group (*p* < 0.05). Meanwhile, the expression of *IL10* was inhibited under different CXCL14 protein concentrations, and the expression of *IL10* (1 μg/mL and 20 μg/mL) was significantly lower than that in the control group (*p* < 0.05) (Figure 3C). The above results were also verified by the detection of inflammatory factors in cell supernatant using ELISA. The results showed that the contents of TNF-α, IL-6 and COX2 in the cell supernatant treated with the CXCL14 protein (1 μg/mL, 10 μg/mL and 20 μg/mL) were significantly higher than those in the control group (*p* < 0.05). However, there was no correlation between the increase in inflammatory factors and the concentration of protein treatment. The contents of TNF-α and COX2 were the highest under the treatment of 20 μg/mL of the CXCL14 protein, while the content of IL-6 was the highest under the treatment of 10 μg/mL of the CXCL14 protein (Figure 3D). These results suggest that the CXCL14 protein might be an important regulator of the M1 polarization of macrophages, and the overexpression of CXCL14 could play a natural immune role by inducing macrophages to M1 polarization.

### 3.4. Effect of CXCL14 Protein on the Activity, Cloning and Migration of HepG2 Cells

**Figure 4:**
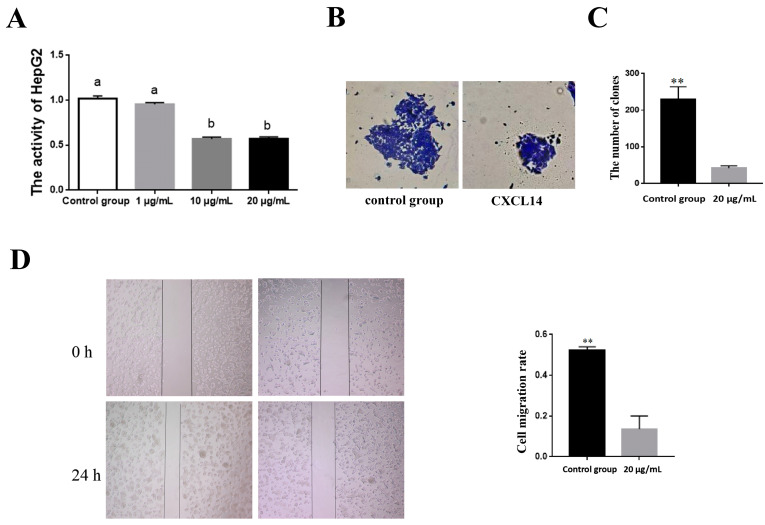
Effect of CXCL14 protein treatment on HepG2. (**A**) HepG2 activity detection. (**B**,**C**) HepG2 clonal formation detection. (**D**) HepG2 migration detection. a,b: Different letters indicate a significant level of difference. Values are expressed as the mean ± SD of *n* = 3; ** *p* < 0.01.

**Figure 5:**
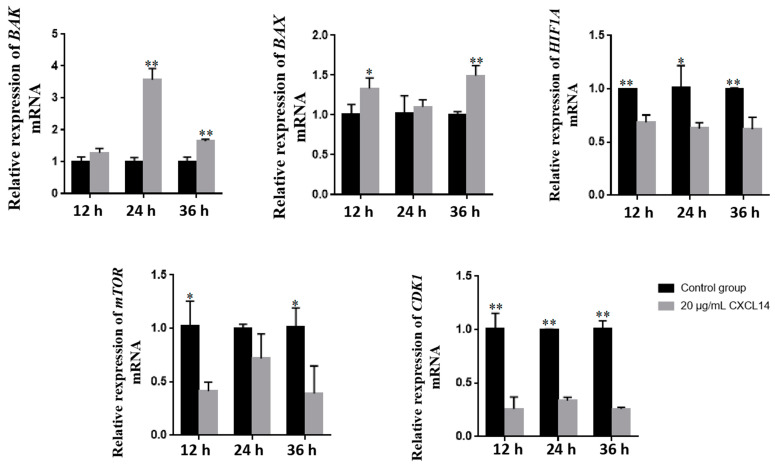
Effect of the CXCL14 protein on the apoptosis gene of HepG2 cells. Values are expressed as the mean ± SD of *n* = 3; * *p* < 0.05, ** *p* < 0.01.

Natural immune cells (such as macrophages) are an important part of the tumor microenvironment. The yak CXCL14 protein can not only activate macrophages to exert natural immunity but also inhibit the activity of HepG2 in a dose-dependent manner, while the activity of HepG2 cells significantly decreased with the increase in protein concentration (*p* < 0.05) (Figure 4A). After being treated with a high concentration of the CXCL14 protein (20 μg/mL), the proliferation and migration ability of HepG2 cells was inhibited, and the number of HepG2 cell clones formed after 24 h was significantly lower than that of the control group (*p* < 0.01) (Figure 4B–D). In addition, with the prolonged treatment of HepG2 cells with 20 μg/mL of the CXCL14 protein, the expression levels of pro-apoptotic genes *BAK* and *BAX* increased significantly, while the expression levels of tumor pro-proliferation genes *HIF1A*, *mTOR* and *CDK1* decreased significantly (Figure 5).

## 4. Discussion

As the largest lymphoid organ in mammals, the spleen not only distributes highly ordered T lymphocytes and B lymphocytes but also contains more macrophages, making it an important organ for the immune response against bacteria and fungi [2]. Early studies have reported that *CXCL14* has been found to be expressed in a variety of immune cells or non-immune cells under different stimuli; therefore, it can play a pro-inflammatory or anti-inflammatory role in the body [4,5]. At present, *CXCL14* has been widely studied in humans and mice but has rarely been reported in yak. Yaks show low sensitivity to the harsh plateau environment and have enhanced viability in harsh environments, which could be related to their unique morphological, physiological and adaptive characteristics. In a previous study, the temporal differential expression analysis of the *CXCL14* gene in the spleen of yaks showed that the *CXCL14* gene increased gradually with age, and its expression reached the highest level in adulthood, indicating that the *CXCL14* gene plays an important role in the development and functional improvement of spleen tissue, which could be related to the chemokines necessary for the development of macrophages [12]. Moreover, with the rapid development of bioinformatics tools, it is very easy and fast to predict the function of proteins. This study predicts that the yak CXCL14 protein has a stable low-complexity domain, which could be the key to its function. At the same time, the CXCL14 protein was also found to have the potential characteristics of antimicrobial peptides (such as an overall positive charge, containing α helix, etc.). The *Escherichia coli* BL21 virulence test also proved that the CXCL14 protein induced by prokaryotic expression in vitro had obvious inhibitory effects on Escherichia coli, indicating that the yak CXCL14 protein showed strong antibacterial activity. 

CXCL14 is not only involved in the inflammatory response of the body but also in the regulation of many biological processes [5]. However, there are few studies on the mechanism of inflammatory response in yak macrophages. In primary yak spleen macrophages, it was found that the CXCL14 protein could stimulate the pro-inflammatory response of cells at a certain concentration and promote the expression of pro-inflammatory factors *IL-1β*, *IL6*, *IL8* and *TNF-α* to significantly increase, while the expression of the anti-inflammatory factor *IL10* decreased significantly. In general, macrophages can often switch between these two states of classically activated macrophages, which secrete pro-inflammatory factors such as IL6, IL8 and TNF-α, and those of vicarious activated macrophages, which produce anti-inflammatory factors such as IL-10 and TGF-β [13]. Therefore, it is speculated that the CXCL14 protein may induce the polarization of macrophage M1 to participate in the innate immune response to prevent the invasion of pathogens and ensure that the body is not damaged; further tests are needed to determine its related mechanism. The phenotypic states of macrophages are closely associated with a variety of diseases, especially cancer. Tumor-associated macrophages are the main immune cells in the tumor microenvironment, and M1 macrophages have strong anti-tumor activity. Increasing the proportion of M1-type macrophages is an important indicator of good prognosis in cancer [14,15]. Although *CXCL14* is a relatively novel gene in the chemokine family, it has long been reported that *CXCL14* is closely related to the occurrence of breast cancer, liver cancer, lung cancer and other cancers [16,17,18,19,20,21,22]. It has been found that *CXCL14* is significantly down-regulated in HPV-positive head/neck cancer and cervical tissue specimens, and restoring the expression of *CXCL14* in oropharyngeal cancer cells could clear tumors in mice [23]. In addition, some studies also found that CXCL14 was significantly down-regulated in HBV-related hepatocellular carcinoma tissues [24]. This study also found that the CXCL14 protein inhibited the growth activity and migration ability of liver cancer cells to a certain extent, which is similar to that reported by others in liver cancer [19]. In addition, the human CXCL14 protein also had similar effects in other cancer cells, such as cervical cancer, esophageal cancer and prostate cancer [25,26,27,28]. Moreover, the apoptotic genes of *BAK* and *BAX* were significantly increased, and proliferation-related genes such as *HIF1A*, *mTOR* and *CDK1* were also inhibited at certain treatment times. This further indicates that the yak CXCL14 protein has a certain promotion effect on the apoptosis of HepG2. However, the pathway of cancer cell apoptosis, when induced by CXCL14, could be related to nuclear apoptosis and mitochondrial apoptosis [29,30,31]. As an exogenous substance, the specific anti-cancer mechanism of the yak CXCL14 protein remains to be further studied. However, this study reveals the potential function of CXCL14 in yak spleen tissue and determines the pro-inflammatory and anti-tumor effects of the yak CXCL14 protein, which could provide some basic data for exploring the immune mechanism of plateau animals and screening anti-cancer drugs derived from animal protein sources in the future.

## 5. Conclusions

In summary, yaks have been used as the best research object to explore the adaptive mechanism of animal bodies to harsh plateau environments. However, there are few reports on how *CXCL14* plays the function of innate immunity in yaks. In this study, the yak CXCL14 protein was analyzed, characterized and overexpressed in vitro, revealing the potential biological function of the CXCL14 protein in yaks to activate innate immunity and inhibit tumorigenesis. This study is helpful to further understand the function of the CXCL14 protein in yaks in vivo and provide certain reference materials for the anti-inflammatory mechanism of yaks.

## Figures and Tables

**Table 1:** Details of the primers used for the analysis of qRT-PCR.

Gene	Primer Sequence (5′−3′)	Tm/°C
*IL-1β*	F: TCACAGCAGCACATCAACAA R: TGTCCTCATCCTGGA AGGT	59
*IL*6	F: ACA ACCACGGCCTTCCCTACTT R: CACGATTTCCCAGAGAACATGTG	60
*IL*10	F: ACCCACTTCCCAGTCGGC R: CGGTTAGCAGTATGTTGTCCA	60
*TNF-a*	F: AAG CCTGTAGCCCACGTCGTA R: GGCACCACTAGTTGGTTGTCTTTG	60
*BAK*	F: CAGGCAGGAGTGCGGAGA R: GCGTCGGTTGATGTCGTC	53
*HIF1A*	F: ATGATACCAACAGTAACCAACC R: TCATAAATTGAGCGGCCTA	60
*CDK1*	F: GGGTCAGCTCGCTACTCAAC R: AAGTTTTTGACGTGGGATGC	60
*BAX*	F: ACGGCAACTTCAACTGGG R: GGGGTGAGGAGGCTTG	53
*mTOR*	F: CTTAGAGGACAGCGGGGAAG R: TCCAAGCATCTTGCCCTGAG	60.0

## Data Availability

Data sharing is not applicable to this article.
